# A Deadline-Aware Scheduling and Forwarding Scheme in Wireless Sensor Networks

**DOI:** 10.3390/s16010059

**Published:** 2016-01-05

**Authors:** Thi-Nga Dao, Seokhoon Yoon, Jangyoung Kim

**Affiliations:** 1 Department of Electrical and Computer Engineering, University of Ulsan, Ulsan 680-749, Korea; daothinga.mta@gmail.com; 2 Department of Computer Science, University of Suwon, Gyeonggi 445-743, Korea; jykim77@suwon.ac.kr

**Keywords:** delay bound, duty-cycled wireless sensor networks, routing protocol, geographic forwarding

## Abstract

Many applications in wireless sensor networks (WSNs) require energy consumption to be minimized and the data delivered to the sink within a specific delay. A usual solution for reducing energy consumption is duty cycling, in which nodes periodically switch between sleep and active states. By increasing the duty cycle interval, consumed energy can be reduced more. However, a large duty cycle interval causes a long end-to-end (E2E) packet delay. As a result, the requirement of a specific delay bound for packet delivery may not be satisfied. In this paper, we aim at maximizing the duty cycle while still guaranteeing that the packets arrive at the sink with the required probability, *i.e.*, the required delay-constrained success ratio (DCSR) is achieved. In order to meet this objective, we propose a novel scheduling and forwarding scheme, namely the deadline-aware scheduling and forwarding (DASF) algorithm. In DASF, the E2E delay distribution with the given network model and parameters is estimated in order to determine the maximum duty cycle interval, with which the required DCSR is satisfied. Each node independently selects a wake-up time using the selected interval, and packets are forwarded to a node in the potential forwarding set, which is determined based on the distance between nodes and the sink. DASF does not require time synchronization between nodes, and a node does not need to maintain neighboring node information in advance. Simulation results show that the proposed scheme can satisfy a required delay-constrained success ratio and outperforms existing algorithms in terms of E2E delay and DCSR.

## 1. Introduction

Wireless sensor networks (WSNs) have been widely used in many applications [1,2], such as military surveillance, agriculture and environment monitoring. A large number of sensors are deployed in a certain region to measure a parameter, and then, they send the data to a sink node. Since sensors are usually provided with a limited battery, reducing energy consumption has attracted a great deal of attention.

Duty cycling is a useful solution to reduce consumed energy and prolong network lifetime. However, sleep scheduling results in a high delay, since a node has to wait for the next hop to be active instead of transmitting data immediately [3]. We note that, in some applications, packets are required to arrive at the sink within a certain period of time. For example, the detection of critical events (e.g., forest fire detection [1]) should be reported to the sink node within a specific delay bound. Thus, in order to respond quickly to important events, a delay-constrained forwarding algorithm with a bounded latency is highly desired in practice.

There have been several studies on both routing and scheduling protocols in order to deal with end-to-end (E2E) delay problems [4,5,6,7,8,9]. However, those existing algorithms require global information [7] (e.g., all one-hop delays and communication links in the network), a high message complexity [6,9] or costly time synchronization between nodes [4,5,8]. Moreover, none of them can estimate the distribution of E2E delay given the network parameters before sensor nodes are deployed in WSNs; *i.e.*, they can predict neither the packet arrival time at the sink nor the fraction of packets that will reach the destination within a specific period of time.

In order to address the limitations of existing algorithms, we propose a delay-constrained forwarding algorithm, namely deadline-aware scheduling and forwarding (DASF), which guarantees that packets arrive at the sink with the required probability, or delay-constrained success ratio (DCSR), while maximizing the duty cycle interval. In DASF, the distribution of E2E delay is estimated as a function of the duty cycle interval and the number of potential forwarders (*m*), whose distribution is also approximated given the network model and parameters. Then, the maximal value of the duty cycle interval is selected with which the required DCSR is satisfied.

Using the selected duty cycle interval, each node under DASF schedules its sleep and wake-up times independently. The sender node forwards the packet to the node that wakes up first among the potential forwarders. DASF does not require time synchronization between sensors in WSNs. Moreover, it enables a human operator to assess E2E delay and perform resource planning (e.g., setting the number of nodes) before actual deployment.

In order to validate the proposed scheme, simulations are performed using various scenarios. Simulation results show that DASF can achieve the required DSCR and outperforms existing algorithms in terms of E2E delay and delay-constrained success ratio (DCSR).

The rest of this paper is organized as follows. Section 2, Section 3 and Section 4 present related work, the problem definition and the network model, respectively. Then, Section 5 describes a delay-constrained forwarding algorithm in detail. Simulation results are presented in Section 6, and Section 7 concludes the paper and discusses future work.

## 2. Related Work

In this section, we present existing studies on routing and scheduling protocols that consider the delay issue in a WSN, and we compare these to our algorithm.

There is synchronous and asynchronous scheduling in duty-cycled WSNs. In synchronous protocols, nodes cooperate with each other and concurrently switch a radio on and off [10,11,12]. For example, Ye *et al.* [10] proposed sensor medium access control (S-MAC) in which a node randomly chooses and announces its awake schedule if it has not received a schedule from its neighboring nodes. When receiving a neighboring node’s schedule, it will follow this schedule, *i.e.*, it synchronizes with other nodes. Unlike synchronous scheduling, in asynchronous protocols [13,14], every node independently chooses its own sleep scheduling. Hence, senders and receivers do not need to wake up at the same time. In order to ensure packet transmission, nodes exchange scheduling information or periodically broadcast beacon messages. For instance, Polastre *et al.* [13] developed berkeley media access control (B-MAC), in which a node that wants to transmit a frame sends a long preamble to make sure that the next hop can hear the packet. Since asynchronous scheduling is much simpler than synchronous, our algorithm uses asynchronous scheduling. However, unlike existing asynchronous scheduling algorithms, we consider a forwarding strategy and delay-bound requirement.

There have been several routing protocols that consider E2E delay in duty-cycled WSNs [4,5,8]. In these protocols, a node makes a forwarding decision based on one-hop delay (*i.e.*, the node selects the next hop that has a low one-hop delay among nodes in the candidate set). For instance, Paruchuri *et al.* [4] proposed random asynchronous wakeup (RAW), in which each node randomly selects its scheduling. Each node also maintains a forwarding candidate set (FCS), which includes nodes closer to the destination than itself by a given threshold distance. In order to forward packets, a sensor node will choose the closest node to the sink among all active nodes in the FCS. If there is no active node in the FCS, the node will wait for the closest node in the FCS to wake up.

In addition, Beraldi *et al.* [5] proposed lukewarm potato forwarding (LPF) in which each node can predict the wake-up time of its neighboring nodes. In LPF, nodes initially build the shortest path tree rooted at the sink. To forward data, a node sends the packet to its parent in the tree if the wait time until its parent wakes up is less than a given threshold value. Otherwise, the packet is transmitted to the node that wakes up earliest among neighboring nodes that are closer to the sink. In order to obtain the wake-up time of neighboring nodes, all nodes use the same pseudo random number generation algorithm, but different seed values. Using a local seed, each node initially generates its active time. When a node wakes up, it broadcasts its local seed, $s_{n}$, and a random number, $S_{n}$. By receiving $s_{n}$ and $S_{n}$, a node is able to predict the next active time of its neighboring node.

Compared to our algorithm, the listed protocols only try to reduce the E2E delay instead of meeting a given delay bound in WSNs. Additionally, they require more information to be exchanged than our algorithm. For instance, in the RAW algorithm, nodes need to exchange beacon messages that include the node ID, clock, schedule, lifespan and location whenever nodes switch on the radio. In the case of LPF, sensor nodes broadcast the node ID, a local seed and a newly-generated random number, $S_{n}$, when they wake up. In contrast, our work only requires nodes to broadcast the node ID and group number. Moreover, RAW, LPF and the gradient-based multi-path routing protocol (GMRP) [8] require time-synchronization [15,16] between nodes, whereas our algorithm does not.

In order to find the path with the least delay, Lai *et al.* [6] introduced a fast time-dependent shortest path algorithm (FTSP) based on the Bellman–Ford algorithm. Messages are exchanged between nodes, and they try to build time-dependent paths with the least delay. However, time and message complexity are high when the total number of nodes increases in WSNs. In particular, when there is a change in the network (*i.e.*, dead nodes), FTSP takes more time to again calculate the path of the least delay. In contrast, our algorithm uses only local information for packet forwarding and, hence, can quickly adapt to the network changes.

There are a few routing protocols on meeting a given delay bound in WSNs [7,9,17]. Wang *et al.* [7] introduced DutyCon to solve the delay problem. Initially, they build *w* disjoint paths, each of which is generated by one of the *w* sources in the network. After that, they apply feedback control theory to adjust the receiver’s sleep interval according to a collected single-hop delay. However, they assume that each node must know global information of its path in the WSN, such as all communication delays of each hop. In contrast, our algorithm does not require global information and nodes do not even maintain scheduling of their neighbors in advance.

Jie Hao *et al.* [18] proposed a localized forwarding algorithm with asynchronous scheduling that does not maintain neighboring node information in advance. First, by assuming that a simple forwarding strategy is used, a source node evaluates a desired mean and variance of the E2E delay that can meet a given delay requirement. After that, in order to satisfy the desired mean of the E2E delay, the authors design a forwarding strategy, such that a relay node makes a forwarding decision as to whether to wait for a better next hop or forward the packet immediately. However, in their work, when a node actually forwards a packet, it only tries to meet the desired mean of the E2E delay without considering variance. As a result, it may not achieve the required DCSR. In contrast, our scheme can achieve the required DCSR by using the optimal duty cycle interval obtained from the estimated distribution of E2E delay.

To sum up, even though there are several studies on scheduling and routing that address delay problems in WSNs, none of them can estimate the distribution of E2E delay before the actual node deployment. However, our algorithm can adjust the duty cycle interval to satisfy delay requirements according to the real routing strategy. In addition, our work only needs local information and does not require time synchronization.

## 3. Problem Definition

In WSNs, an application often has a specific requirement for packet latency. For example, packets may be required to reach the sink within a certain delay limitation with a given probability. Let random variable *Z* denote the E2E delay. Then, *Z* can be considered as the sum of the one-hop delays on the path from the source to the sink. Let *Y* denote a vector in which each element is the one-hop delay of a path from the source to the sink. If *h* is the hop count, $Y=\left\{Y_{1},Y_{2},...,Y_{h}\right\}$ with $Y_{q}$ as the one-hop delay at the *q*-th hop (1≤q≤h). Therefore, *Z* can be expressed as a function of *Y*:

(1) $$Z=\sum_{q=1}^{h}\left(Y_{q}\right)$$

If we apply duty cycling in WSNs, a one-hop delay will be affected by sleep scheduling (the duty cycle interval and active time) and network parameters. $Y_{q}$ can be represented as a function of the duty cycle interval and other parameters:

(2) $$Y_{q}=f\left(T,\phi_{q}\right)$$

where $\phi_{q}$ denotes the set of network parameters, such as the number of neighboring nodes, the total area and the communication range of a node.

Then, the optimization problem in this paper can be formulated as follows. Maximize duty cycle interval *T* with given network parameters and the E2E delay constraint that a packet should reach the sink by delay bound *ξ* with given success ratio $p_{s}$. That is,

(3) $$\begin{array}{c} \mathrm{maximize}\;T\\ \mathrm{subject}\mathrm{to}:P\left(Z<\xi \right)\geq p_{s} \end{array}$$

where $p_{s}$ is the required DCSR.

## 4. Network Model

**Figure 1:**
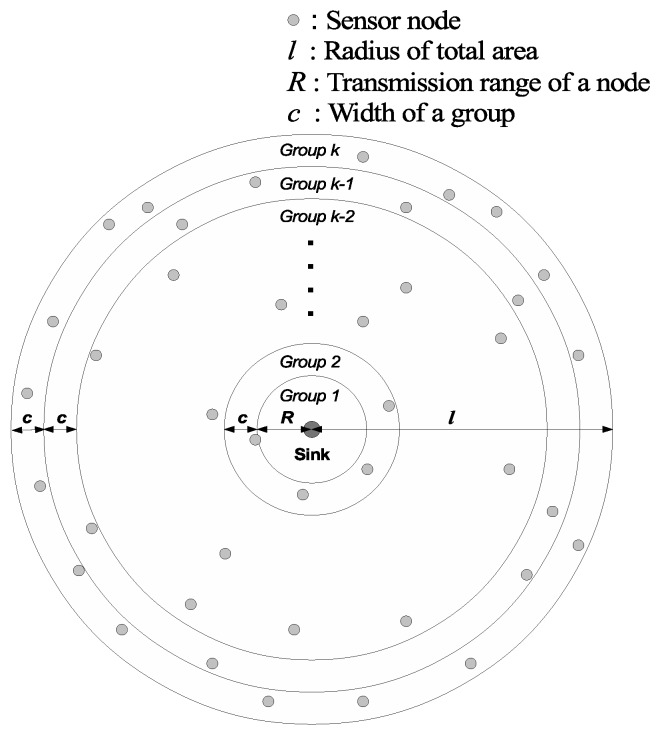
Node deployment in a WSN.

We assume that sensors are deployed randomly in a circular area around the sink node with radius *l* and each node knows its position by using localization techniques [19,20]. In this paper, we denote *N* and *R* as the total number of nodes and the transmission range of a sensor node, respectively. Nodes are partitioned into multiple groups according to the distance between node and sink. Let *c* denote the width of a group. Node *A* obtains its group number using $d_{A}$, the distance between node *A* and the sink. Figure 1 illustrates node deployment in the total area. Node *A* belongs to Group 1 if $0\leq d_{A}\leq R$ or if it can transmit packets directly to the sink. Node *A* belongs to group *j* if $d_{A}$ satisfies:

(4) $$R+c\left(j-2\right)<d_{A}\leq R+c*\left(j-1\right)$$

where $0\leq d_{A}\leq l$. The total number of groups, denoted by *k*, is calculated as follows:

(5) $$k=\left\lceil \frac{l-R}{c}\right\rceil +1$$

In order to reduce energy consumption, duty cycling is considered in the network. We denote *T* and $T_{a}$ as the duty cycle interval and the active period, respectively. Sensor nodes independently choose a random wake-up time and stay awake for $T_{a}$. Then, they switch the radio off for the rest of time of the duty cycle interval. Whenever nodes switch the radio on, they broadcast beacon messages that include only the node ID and group number. Time synchronization is not required between nodes.

## 5. A Deadline-Aware Scheduling and Forwarding Scheme

In order to solve the problem in Equation (3), we need to obtain the distribution function of E2E delay of a packet in the WSN. Since E2E delay is the sum of one-hop delays, as in Equation (1), we estimate the one-hop delay distribution. Nodes are deployed randomly with a uniform distribution, and each relay node makes a forwarding decision independently. In a dense network, each node has a similar number of neighboring nodes. Note that one-hop delay random variables can be considered to have the same distribution function. Therefore, in order to approximate the E2E delay distribution, we apply the central limit theorem for a long path, *i.e.*, the E2E delay is approximated by the normal distribution function where the mean and variance of E2E delay are the sum of the mean and variance of one-hop delays, respectively.

In this section, we first describe the proposed delay-constrained forwarding algorithm and then approximate the E2E delay distribution using the duty cycle interval and network parameters. Then, we show that with the given network parameters, it is possible to adjust the duty cycle interval to satisfy the delay bound with a desired delay-constrained success ratio.

### 5.1. Group-Based Forwarding

To begin with, we consider node *A* in group *j* and assume that it has *m* potential forwarder(s), whose group ID is less than node *A*’s. The transmitter selects the first active node among *m* potential forwarders. Assume that node *A* has three potential forwarders (e.g., nodes *B*, *C* and *D*). Suppose that node *A* has a packet to send to the sink at time $t_{0}$. On receiving a beacon message, node *A* knows that a sender of this message is active. If node *A* receives a beacon message first broadcast by *C* among three potential candidates, node *A* will select node *C* as the next forwarder.

Let random variable *M* denote the number of potential forwarders of a node in group *j*. Since one-hop delay and E2E delay depend on the value of *M*, we need to estimate the distribution of *M*. After that, based on the forwarding algorithm, we present the distribution of the one-hop delay and E2E delay.

### 5.2. The Distribution of *M*

**Figure 2:**
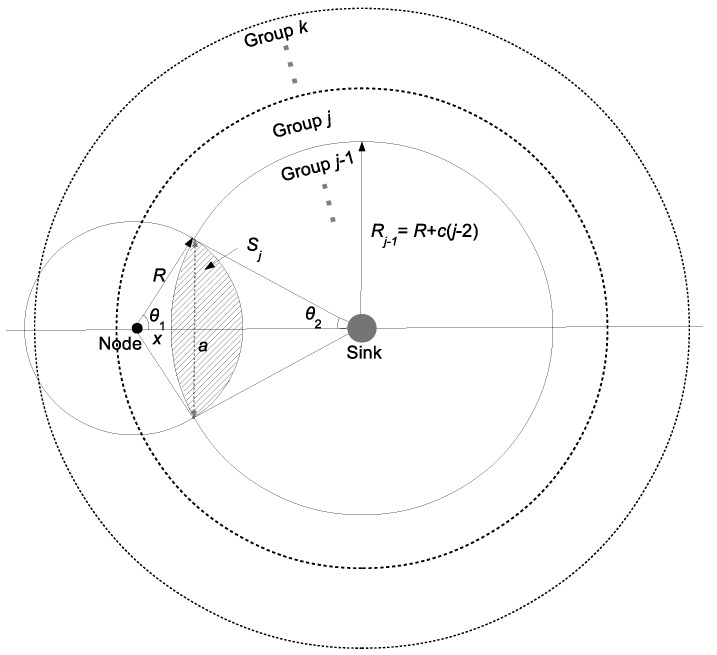
Intersection area.

In order to estimate E2E delay’s distribution, we estimate the distribution of random variable *M* with given network parameters and the sender’s position. First, we define node density $\rho =\frac{N}{S}$ where *N* is the total number of nodes in the WSN and *S* is the total area size. As shown in Figure 2, let random variable *X* denote the distance between the sender (group *j*) and the boundary of group j-1; so that *X* has a uniform distribution with the range 0≤X≤c, where *c* is the width of a group. $S_{j}$ is a forwarding area over which the next hop has to deploy; in other words, $S_{j}$ is the intersection area between a communication area of the sender and a circular area of the group j-1 that has a radius $R_{j-1}=R+c\left(j-2\right)$. $S_{j}$ is also the random variable that depends on variable *X* and the group ID of the node. Figure 2 illustrates forwarder area $S_{j}$ as the dashed area.

According to Bettstetter [21], the number of nodes in certain area $A_{0}$ follows a Poisson distribution and can be expressed as follows:

$$P\left(n_{0}\right)=\frac{{\left(\rho A_{0}\right)}^{n_{0}}}{n_{0}!}e^{-\rho A_{0}}$$

Hence, the probability of finding *m* nodes in intersection area $S_{j}$ is expressed as follows:

(6) $$P_{j}\left(M=m\right)=P_{j}\left(m\right)=\frac{{\left(\rho S_{j}\right)}^{m}}{m!}e^{-\rho S_{j}}$$

Let $S_{j,1}$ denote the circular sector of the communication range of the node with radius *R* and central angle $2\theta_{1}$, $S_{j,2}$ denote the circular sector of the circle of group j-1 with radius $R_{j-1}$ and central angle $2\theta_{2}$ and $S_{j,3}$ denote the kite area with diagonals *a* and $\left(x+R_{j-1}\right)$. According to Figure 2, we calculate $S_{j}$ as follows:

(7) $$\begin{array}{c} S_{j}=S_{j,1}+S_{j,2}+S_{j,3}\\ \;\;\;\;=\int_{-\theta_{1}}^{\theta_{1}}\int_{0}^{R}rdrd\theta +\int_{-\theta_{2}}^{\theta_{2}}\int_{0}^{R_{j-1}}rdrd\theta -a\left(x+R_{j-1}\right) \end{array}$$

where $\theta_{1}=\arccos\left(\frac{R^{2}+{\left(x+R_{j-1}\right)}^{2}-R_{j-1}^{2}}{2R(x+R_{j-1})}\right)$, $\theta_{2}=\arccos\left(\frac{R_{j-1}^{2}+{\left(x+R_{j-1}\right)}^{2}-R^{2}}{2R_{j-1}\left(x+R_{j-1}\right)}\right)$ and $a\;=\;2R\sin\theta_{1}$.

**Figure 3:**
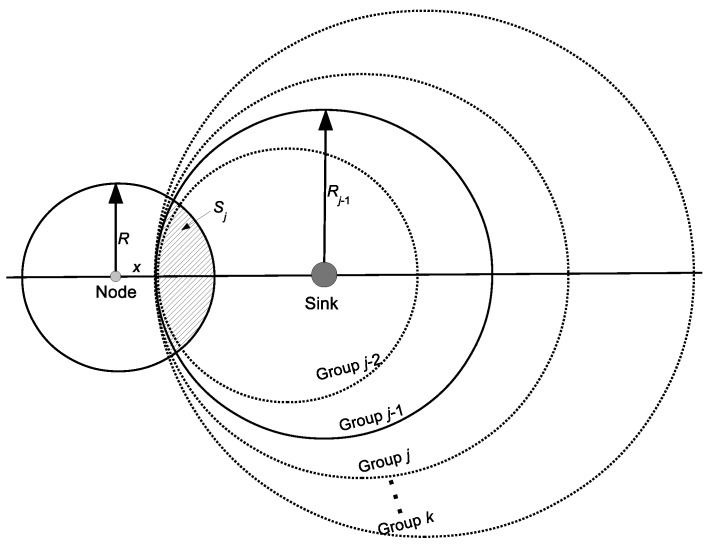
Intersection area with different groups.

In order to compare the intersection areas with different values of group ID, we can redraw the intersection areas as shown in Figure 3. We keep the position of the circle of group *j*. When group ID $j_{0}$ is greater than *j*, the circle of group $j_{0}$ is shifted in the direction from the node to the sink by $c(j_{0}-j)$. When the group ID $j_{0}$ is less than *j*, the circle of group $j_{0}$ is shifted in the direction from the sink to the node by $c(j-j_{0})$. All of $S_{j}$ are intersected at the point where the distance to the node is *x*. As can be seen from Figure 3, with the same value of *x*, the intersection area of $S_{j}$ between the communication range of the node and group *j*’s circle is interior to that of $S_{j+1}$ and exterior to that of $S_{j-1}$, *i.e.*, $S_{j+1}>S_{j}>S_{j-1}$.

Equation (6) defines $P_{j}\left(m\right)$ with a certain value of *X*. Hence, according to Bayes’ theorem, the probability that a node in group *j* has *m* potential forwarders is as follows:

(8) $$\begin{array}{c} P_{j}\left(m\right)=\int_{0}^{c}\frac{{\left(\rho S_{j}\right)}^{m}}{m!}e^{-\rho S_{j}}\times P\left(X=x\right)dx\\ \;\;\;\;\;\;\;\;\;\;=\frac{1}{c}\int_{0}^{c}\frac{{\left(\rho S_{j}\right)}^{m}}{m!}e^{-\rho S_{j}}dx \end{array}$$

where $S_{j}$ is calculated in Equation (7) as a function of *x*, group ID *j* and other network parameters. From Equation (8), $P_{j}\left(m\right)$ depends on node density, intersection area $S_{j}$ and the group width.

### 5.3. One-Hop Delay Distribution

In this subsection, we describe the estimation of the one-hop delay distribution of node *A* in an arbitrary group *j* (2≤j≤k). Let *m* denote the number of potential forwarders of node *A* (1≤m<N). Node *A* selects the next hop among *m* potential forwarder(s). Let random variable $D_{i}$ denote the wake-up time of the *i*-th node among *m* potential forwarder(s) (1≤i≤m). Since sensor nodes randomly and independently choose the wake up time, $D_{i}$ are independent and identical variables. The distribution function of $D_{i}$ is $F_{D_{i}}\left(d_{i}\right)=P\left(D_{i}\leq d_{i}\right)=\frac{d_{i}}{T}$ if $0\leq d_{i}\leq T$. Let random variable $Y_{j}$ denote one-hop delay of the packet at group *j*, then $Y_{j}$ will be defined as follows: $Y_{j}=min\left(D_{1},D_{2},...,D_{m}\right)$. Let $P(Y_{j}<y_{j}|m)$ define the probability that one-hop latency is shorter than $y_{j}$ given *m*, *i.e.*, the probability that there exists at least one $D_{i}<y_{j}$ if the node has *m* potential forwarder(s). Then, the distribution function $F(y_{j}|m)$ of $y_{j}$ given *m* is expressed as:

(9) $$\begin{array}{c} F\left(y_{j}|m\right)=P\left(Y_{j}\leq y_{j}|m\right)=1-\text{the}\;\text{probability}\;\text{that}\;\text{all}\;D_{i}>y_{j}\\ \;\;\;\;\;\;\;\;\;\;\;\;\;={\left.1-\prod_{i=1}^{m}\left(1-F_{D_{i}}\left(d_{i}\right)\right)\right|}_{d_{i}=y_{j}}\\ \;\;\;\;\;\;\;\;\;\;\;\;\;=1-{\left(1-\frac{y_{j}}{T}\right)}^{m} \end{array}$$

Now, the distribution function $F(y_{j})$ can be obtained using Bayes’s theorem, that is:

(10) $$\begin{array}{c} F\left(y_{j}\right)=\sum_{m=0}^{N-1}P_{j}\left(m\right)F\left(y_{j}|m\right)\\ \;\;\;\;\;\;\;\;\;=\sum_{m=0}^{N-1}P_{j}\left(m\right)\left[1-{\left(1-\frac{y_{j}}{T}\right)}^{m}\right] \end{array}$$

where *N* is the total number of nodes in the given area and $P_{j}\left(m\right)$, calculated in Equation (8) is the probability that node *A* in group *j* has *m* potential forwarders. From Equation (10), we calculate the probability density distribution $f(y_{j})$ as follows:

(11) $$\begin{array}{c} f\left(y_{j}\right)=\frac{dF(y_{j})}{dy_{j}}\\ \;\;\;\;\;\;\;\;\;=\sum_{m=1}^{N-1}P_{j}\left(m\right)\frac{m}{T}{\left(1-\frac{y_{j}}{T}\right)}^{m-1} \end{array}$$

Because each node randomly chooses the active time in the duty cycle interval period, the one-hop delay should be less than the duty cycle interval or $0\leq Y_{j}\leq T$. Thus, the mean ($\mu_{j}$) and variance ($\sigma_{j}$), respectively, of one-hop delay $Y_{j}$ are calculated as follows:

(12) $$\begin{array}{c} \mu_{j}=\int_{0}^{T}y_{j}f\left(y_{j}\right)\;dy_{j}\\ \;\;\;\;=\int_{0}^{T}y_{j}\left(\sum_{m=1}^{N-1}P_{j}\left(m\right)\frac{m}{T}{\left(1-\frac{y_{j}}{T}\right)}^{m-1}\right)\;dy_{j}\\ \;\;\;\;=\sum_{m=1}^{N-1}P_{j}\left(m\right)\frac{T}{m+1}\\ \;\;\;\;=\alpha_{j}T \end{array}$$

(13) $$\begin{array}{c} \sigma_{j}^{2}=\int_{0}^{T}{\left(y_{j}-\mu_{j}\right)}^{2}f\left(y_{j}\right)\;dy_{j}\\ \;\;\;\;=\int_{0}^{T}y_{j}^{2}f\left(y_{j}\right)\;dy_{j}-\mu_{j}^{2}\\ \;\;\;\;=\sum_{m=1}^{N-1}P_{j}\left(m\right)\frac{2T^{2}}{\left(m+1)(m+2\right)}-\mu_{j}^{2}\\ \;\;\;\;=\beta_{j}T^{2}-\alpha_{j}^{2}T^{2} \end{array}$$

where $\alpha_{j}=\sum_{m=1}^{N-1}P_{j}\left(m\right)\frac{1}{m+1}$ and $\beta_{j}=\sum_{m=1}^{N-1}P_{j}\left(m\right)\frac{2}{\left(m+1)(m+2\right)}$. Note that $P_{j}\left(m\right)$ is a function calculated in Subsection 5.2. Then, from Equations (12) and (13), the mean and variance of one-hop delay $Y_{j}$ can be obtained as a function of the duty cycle interval with given network parameters.

### 5.4. E2E Delay Distribution

In this section, we evaluate the E2E delay distribution. Let random variable *Z* denote E2E delay of the node in group *k*. Since packets are forwarded to the first active node among *m* potential forwarders that have a smaller group ID than the sender, in the worst case, E2E delay can be approximated as the sum of k-1 one-hop delays:

(14) $$Z=Y_{k}+Y_{k-1}+...+Y_{2}$$

where random variable $Y_{j}$ is the one-hop delay of the node in group *j*. Note that $Y_{1}$ is zero, since the sink is always active. In Subsection 5.2, we show that $S_{2}<S_{3}<...<S_{k}$ with the same value of *x*. Since $Y_{2}>Y_{3}>...>Y_{k}$, then $Z<Z^{\prime }$ with $Z^{\prime }=\left(k-1\right)Y_{2}$. If $Z^{\prime }$ satisfies the delay requirement, E2E delay *Z* satisfies, as well. Let $\mu_{j}$ and $\sigma_{j}^{2}$ be the mean and variance, respectively, of one-hop delay $Y_{j}$. Furthermore, let *μ* and $\sigma^{2}$ be the mean and variance, respectively, of $Z^{\prime }$. Thus, $\mu =\left(k-1\right)\mu_{2}$ and $\sigma^{2}=\left(k-1\right)\sigma_{2}^{2}$.

Therefore, if the value of *k* is sufficiently large, the central limit theorem states that the distribution function $F(z^{\prime })$ approaches a normal distribution with the same mean and variance:

(15) $$F\left(z^{\prime }\right)≃G\left(\frac{z^{\prime }-\mu }{\sigma }\right)=G\left(\frac{z^{\prime }-\left(k-1\right)\mu_{2}}{\sqrt{\left(k-1\right)\sigma_{2}^{2}}}\right)$$

Then, $f(z^{\prime })$, the probability density function of $Z^{\prime }$, is as follows:

(16) $$f\left(z^{\prime }\right)≃\frac{1}{\sqrt{2\pi \sigma^{2}}}e^{-\frac{{\left(z^{\prime }-\mu \right)}^{2}}{2\sigma^{2}}}=\frac{1}{\sqrt{2\pi \left(k-1\right)\sigma_{2}^{2}}}e^{-\frac{{\left(z^{\prime }-\left(k-1\right)\mu_{2}\right)}^{2}}{2\left(k-1\right)\sigma_{2}^{2}}}$$

where $\mu_{2}$ and $\sigma_{2}$ are calculated using Equations (12) and (13), respectively. From Equation (16), $f(z^{\prime })$ depends on the group ID of sender node, $\mu_{2}$, and $\sigma_{2}$. As stated in the previous subsection, the mean and variance of one-hop delay are dependent on duty cycle interval; thus, the distribution function of $Z^{\prime }$ depends on duty cycle interval *T*. This means that by adjusting duty cycle interval *T*, we can keep E2E delay under the delay bound with a given DCSR. If random variable $Z_{0}=\frac{Z^{\prime }-\mu }{\sigma }=\frac{Z^{\prime }-\left(k-1\right)\alpha_{2}T}{\sqrt{\left(k-1\right)\left(\beta_{2}-\alpha_{2}^{2}\right)T^{2}}}$, then $Z_{0}$ has a standard normal distribution.

Now, we substitute $Z^{\prime }$ as the delay bound *ξ*; in order to satisfy the delay requirement with a given probability, the duty cycle interval should be expressed as follows:

(17) $$T\leq \frac{\xi }{\left(k-1\right)\alpha_{2}+\sqrt{\left(k-1\right)\left(\beta_{2}-\alpha_{2}^{2}\right)}Z_{0}}$$

where $Z_{0}$ is extracted from the standard normal cumulative distribution function table with a given probability and $Z_{0}$ stands for required DCSR $p_{s}$. Let $T_{max}=\frac{\xi }{\left(k-1\right)\alpha_{2}+\sqrt{\left(k-1\right)\left(\beta_{2}-\alpha_{2}^{2}\right)}Z_{0}}$ denote the maximum duty cycle interval that satisfies the given delay requirement, *i.e.*, at least $p_{s}$% of the packets are forwarded to the sink within delay bound *ξ*. As can be seen from Equation (17), $T_{max}$ should be a function of delay bound, node density and the required DCSR. In Section 6, using the value of $T_{max}$, we will present network performance in DASF under different network parameters and delay requirement.

## 6. Performance Study

In this section, we present the simulation setups and results to validate the proposed algorithm. Network Simulator 2 (NS2) was used for the simulations.

System parameters of DASF were set as follows. Sensor nodes are deployed randomly with a uniform distribution in a circular area with a radius of 300 m, where the sink is placed at the center of the region. We select the communication range of the Mica node family [22] in the simulation. The communication range of the sensor node and the group width are set to R=75 and c=37.5 m, respectively. Therefore, according to Equation (5), the total number of groups is k=7. In the simulation, the number of hops is six. In addition, the data packet and beacon message size are set to 46 and six bytes, respectively. There are four source nodes, and the default value of the event rate is 0.5 pkt/s. Note that sensor nodes randomly generate their own data. Duty cycling and simulation time are set to 6% and 3000 s. Table 1 summarizes the system parameters in the simulation. Table 2 shows the ranges of parameter values and their default values. Note that node density value means the number of nodes over a 60 × 60 m${}^{2}$ area. In order to obtain fair results, we ran the simulations with six different seed values and took the average value. In the simulation, nodes in Group 7 are randomly selected as the source nodes.

We analyze network performance in terms of packet delivery ratio (PDR), average E2E delay and DCSR. We first validate whether our algorithm can adjust the duty cycle interval to satisfy the required DCSR and delay bound, as described in Subsection 6.1. Then, we compared the performance between the proposed algorithm, RAW [4] and LPF [5] with a one-parent tree using different values of node density, event rate and the number of sources. Note that although the existing algorithms consider the delay problem, none of them, including RAW and LPF, can adjust the duty cycle interval to meet the delay requirement. With DASF, the optimal duty cycle interval can be obtained given different delay requirements and network parameters, such as delay bound, DCSR and node density. Therefore, in the simulations, the duty cycle interval that was calculated based on our algorithm was applied to both RAW and LPF.

**Table 1:** Simulation setup.

Radius of total circular area	300 m
Transmission range of a node	75 m
Group width	37.5 m
Data packet size	46 bytes
Beacon message size	6 bytes
Duty cycling	6%
Simulation time	3000 s

**Table 2:** Network parameters. DCSR, delay-constrained success ratio.

Parameter	Range	Default Value
Required DCSR (%)	{80 85 90 95 97}	95
Delay bound (s)	{10 20 30 40 50}	20
Node density	{3 4 6 8 10 12}	8
Event rate (packets/s)	{0.1 0.5 1.0 1.5 2.0 2.5 3.0}	0.5
The number of sources	{1 2 4 8 12 16 20}	4

According to the simulation results, the delay requirement can be satisfied by our algorithm by changing the duty cycle interval according to the given delay bound, the required DCSR and node density. Moreover, DASF can achieve a low delay compared to LPF and RAW algorithms.

### 6.1. Adaptation to Requirement Changes

#### 6.1.1. Effects of Required DCSR Changes

**Figure 4:**
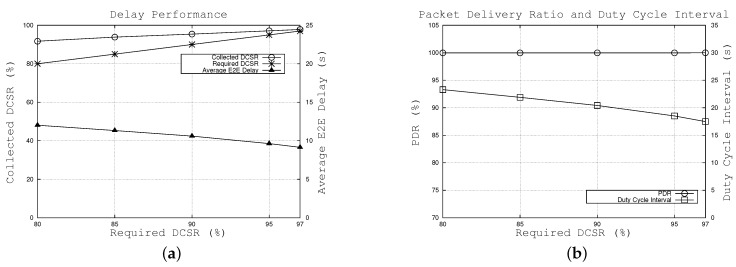
Effects of required DCSR. (**a**) effects on achieved DCSR and average E2E delay, (**b**) effects on packet delivery ratio and duty cycle interval.

Figure 4 illustrates the effect of the required DCSR, $p_{s}$, which varies from 80% to 97%. Figure 4a shows the collected DCSR for different required DCSRs and also the average E2E delay when using the proposed algorithm. The results show that the required delay-constrained success ratio is satisfied and the collected DCSR is higher than the required DCSR. This is attributed to the fact that the duty cycle interval is calculated in the worst case in which the transmission hop is six and the set of random variables $S_{j}$ is identically distributed. For example, when the requirement is set to 80%, 91.7% of the packets can reach the sink within 20 s. In addition, we can see that the gap between the collected and required DCSR tends to become smaller when the required DCSR increases, as shown in Figure 4a.

Moreover, as shown in Figure 4b, the calculated duty cycle interval decreases as the required DCSR increases. This means nodes wake up more frequently; in other words, packets tend to be forwarded earlier. Since other parameters remain unchanged (e.g., node density, delay requirement), the duty cycle interval drop results in the decreased average E2E delay, as shown in Equation (12). For example, when the required DCSR varies from 80% to 97%, the duty cycle interval and the average E2E delay gradually fall from 23.29 s to 17.49 s and 12.01 s to 9.14 s, respectively. Figure 4b also shows that our algorithm can achieve a high packet delivery ratio, approximately 100% over the range of required DCSR. From Figure 4, the results also indicate that the duty cycle interval plays a key role in the E2E delay. Thus, we estimated packet delay as a function of duty cycle interval in the proposed algorithm.

#### 6.1.2. Effects of Delay Bound

**Figure 5:**
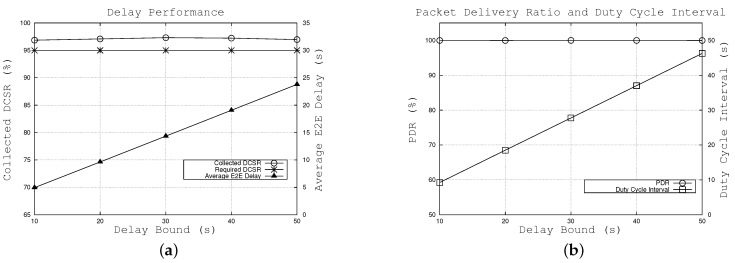
Effects of the delay bound. (**a**) effects on achieved DCSR and average E2E delay, (**b**) effects on packet delivery ratio and duty cycle interval.

In order to examine the effects of delay bound, we kept all parameters as default values, except for the delay bound *ξ*, which varies between 10 s and 50 s. In this scenario, the required DCSR, $p_{s}$, is set to 95%, which means at least 95% of the packets are required to reach the sink within the delay bound. Figure 5a shows that DASF meets the required DCSR (95%) with different delay bounds, and the difference between the collected and required DCSRs is always similar. For instance, approximately 97% of the packets can be forwarded to the sink under different delay bound values. The reason is as follows. In this subsection, we keep the required DCSR unchanged over different values of the delay bound. In order to maintain the unchanged DCSR $p_{s}=\frac{\xi -\mu }{\sigma }$, the average *μ* and variance *σ* of $Z^{\prime }$ should be changed. Since *μ* and *σ* are a function of the duty cycle interval *T*, as shown in Subsection 5.3, the algorithm tunes the value of *T*, such that $p_{s}$ remains immutable.

Figure 5b illustrates that the calculated duty cycle interval tends to increase when the delay bound increases. As a result, nodes wake up less frequently, which leads to a longer E2E delay. As shown in Equation (12), the duty cycle interval growth causes the increased average E2E delay of the packets. For instance, when the delay bound is set from 10 s to 50 s, the calculated duty cycle interval linearly increases from 9.2 s to 46.2 s and the collected average E2E delay gradually increases from 4.9 s to 23.8 s. In terms of PDR, Figure 5b shows that our algorithm can achieve a high packet delivery ratio, made up of nearly 100% over different values of delay bound.

### 6.2. Performance Comparison with Other Algorithms

In this subsection, we consider the network performance of our algorithm, LPF and RAW under different values of node density, event rate and the number of sources. Note that the calculated duty cycle interval obtained from our algorithm is used for the LPF and RAW algorithms, since they are not able to find the optimal duty cycle interval.

**Figure 6:**
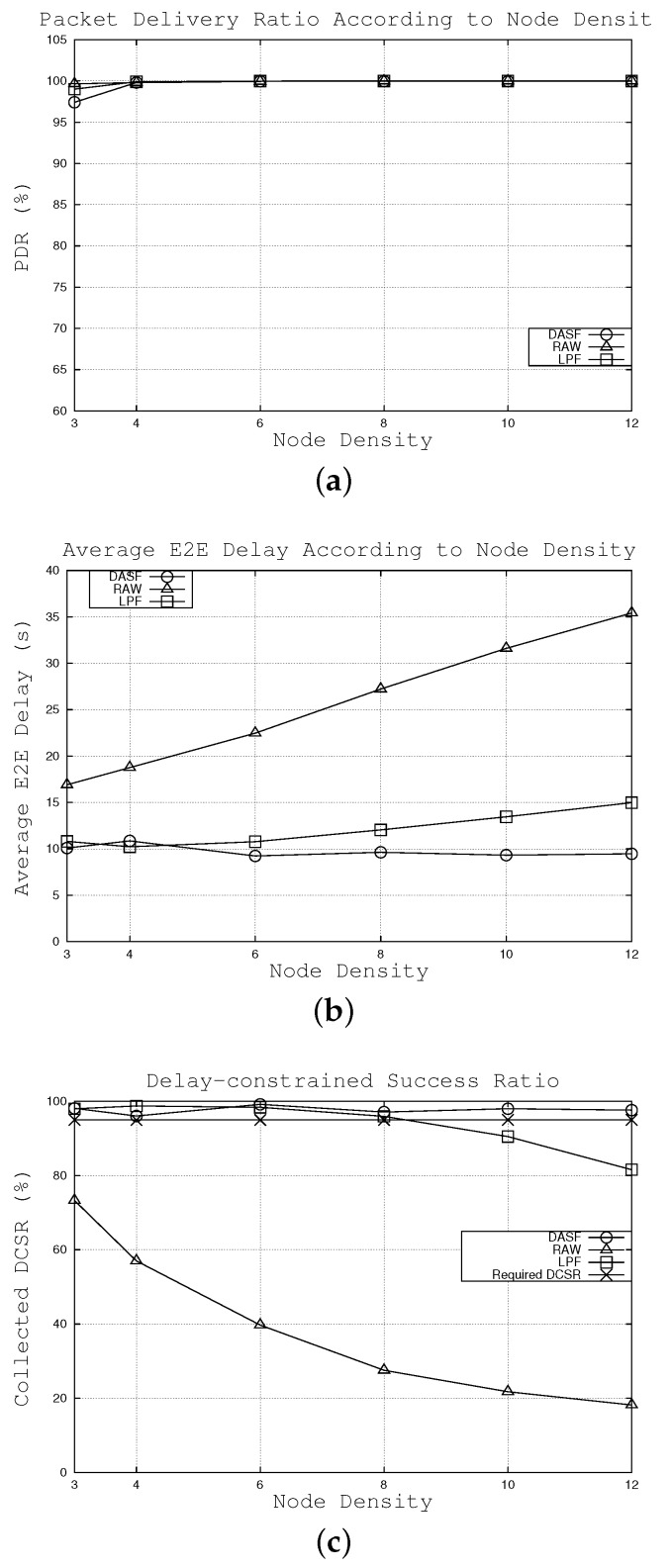
Effects of the node density. (**a**) effects on packet delivery ratio, (**b**) effects on average E2E delay, (**c**) effects on collected DCSR.

#### 6.2.1. Effects of Node Density

The effects of node density on network performance are examined for DASF, LPF and RAW algorithms. The node density varies from three to 12 nodes over a 60 × 60 m${}^{2}$ area, while other parameters keep default values. The calculated duty cycle interval values, using Equation (17), are {8.72, 10.36, 14.23, 18.51, 22.97, 27.51} s when the node density is {3, 4, 6, 8, 10, 12}, respectively. As shown in Figure 6a, the packet delivery ratio of three algorithms is high over different values of node density.

As shown in Figure 6b, while the E2E delay of DASF does not change much, that of RAW keeps increasing. With RAW, while a node forwards a packet, if there is no active node in the forwarding candidate set, the sender will wait until the closest node to the sink among candidate nodes wakes up. Therefore, the E2E delay increases with an increase of the duty cycle interval. Nevertheless, in DASF and LPF, the node chooses the first node to wake up among the potential receivers. Thus, as can be seen from Figure 6b, DASF and LPF witness relatively consistent average E2E latency. Note that DASF achieves the lowest E2E delay in most cases. For example, when the node density is 10, packets are forwarded to the sink within 9.32 s on average under the DASF scheme. Meanwhile, the average E2E delay is 13.45 and 31.61 s under the LPF and RAW algorithms, respectively.

As shown in Figure 6c, only our algorithm can satisfy the delay requirement over the range of node densities. LPF can guarantee the delay bound until the node density is eight. When the node density is greater than eight, LPF’s performance is under the requirement. For example, when the node density is 10, under DASF, 98.0% of the packets can reach the sink within 20 s compared to 90.5 and only 21.7% with the LPF and RAW algorithms, respectively. Additionally, our algorithm allows the operator to predict the number of needed nodes over a certain area in order to meet the delay requirement.

#### 6.2.2. Effects of Event Rate

**Figure 7:**
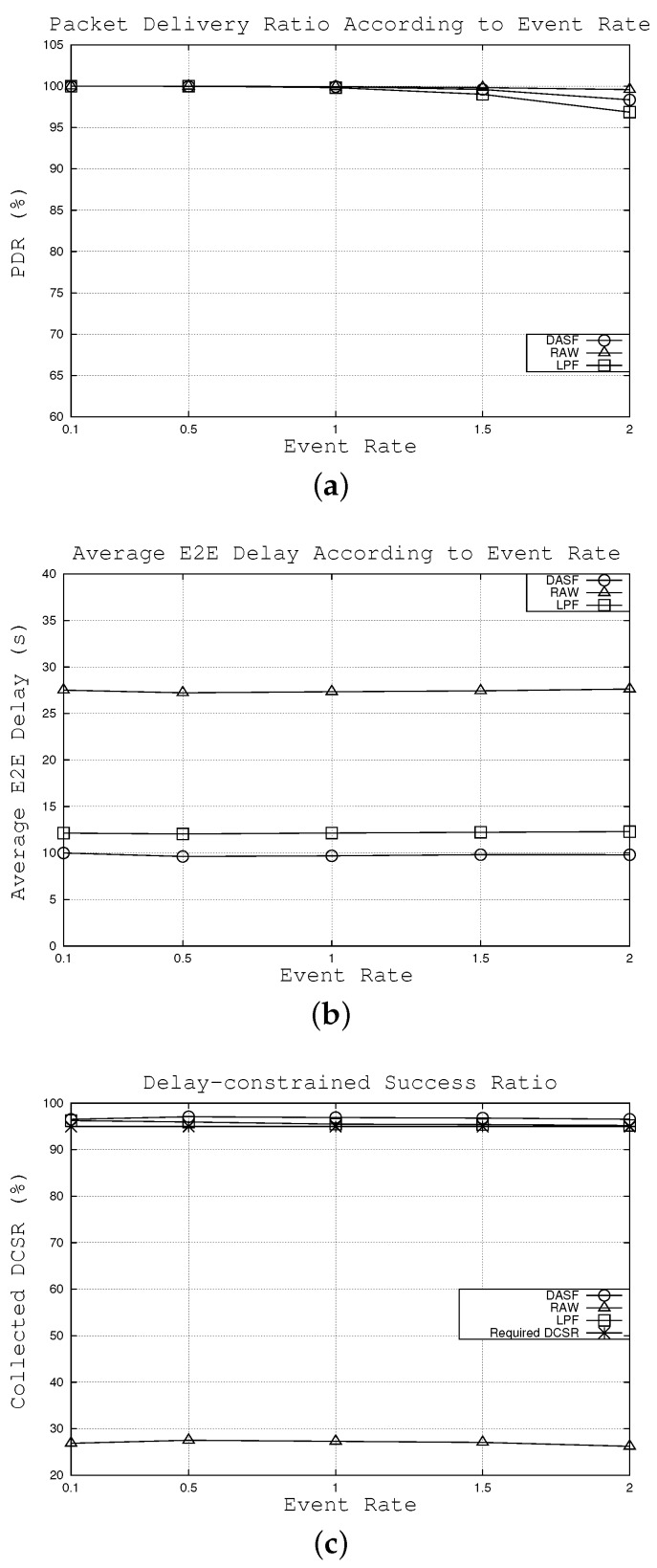
Effects of the event rate. (**a**) effects on packet delivery ratio, (**b**) effects on average E2E delay, (**c**) effects on collected DCSR.

In this subsection, we compare the network performance of the three algorithms for different values of event rate, which change from 0.1 to two packets per second. According to the network parameters, such as node density, delay bound and delay-constrained success ratio, the duty cycle interval is calculated as 18.51 s by using the proposed algorithm. As seen in Figure 7a, the packet delivery ratio tends to decrease when the event rate increases. However, LPF shows the strongest decline, followed by the DASF and RAW algorithms, because the number of hops under RAW tends to be the smallest compared to LPF and DASF. For instance, when event rate is two packets per second, 99.58% of the packets can be delivered successfully to the sink under RAW followed by 98.34% and 96.85% under DASF and LPF algorithms, respectively. Even though the packet delivery ratio of DASF is slightly lower than that of the RAW algorithm, it is still acceptable for most scenarios in WSNs.

As far as the latency is concerned, Figure 7b shows that the averages of E2E delay remain relatively consistent under the three considered algorithms. The reason is that all network parameters except event rate are set to default values. Compared to the results in Subsection 6.2.1, we can clearly see that average E2E delay depends more on node density than event rate, *i.e.*, node density has a great deal of effect on the average E2E delay. In addition, our algorithm can reach the lowest average E2E delay among the three algorithms. For example, when event rate is set to 1.5 packets per second, packets are forwarded to the sink, on average, in 9.8 s under DASF compared to 12.2 s and 27.4 s in cases of LPF and RAW algorithms, respectively.

Figure 7c illustrates the proportion of packets that can satisfy the constraint that E2E delay is under the delay bound (20 s) with the required DCSR (95%). While DASF and LPF can meet the required DCSR, in contrast, RAW does not satisfy. Note that the collected DCSR of our algorithm stays higher than that of LPF. For instance, when the event rate is 1.0 packets per second, 96.9% of the packets can reach the sink successfully within the delay bound compared to 95.5% and 27.3% with LPF and RAW algorithms, respectively.

#### 6.2.3. Effects of the Number of Sources

**Figure 8:**
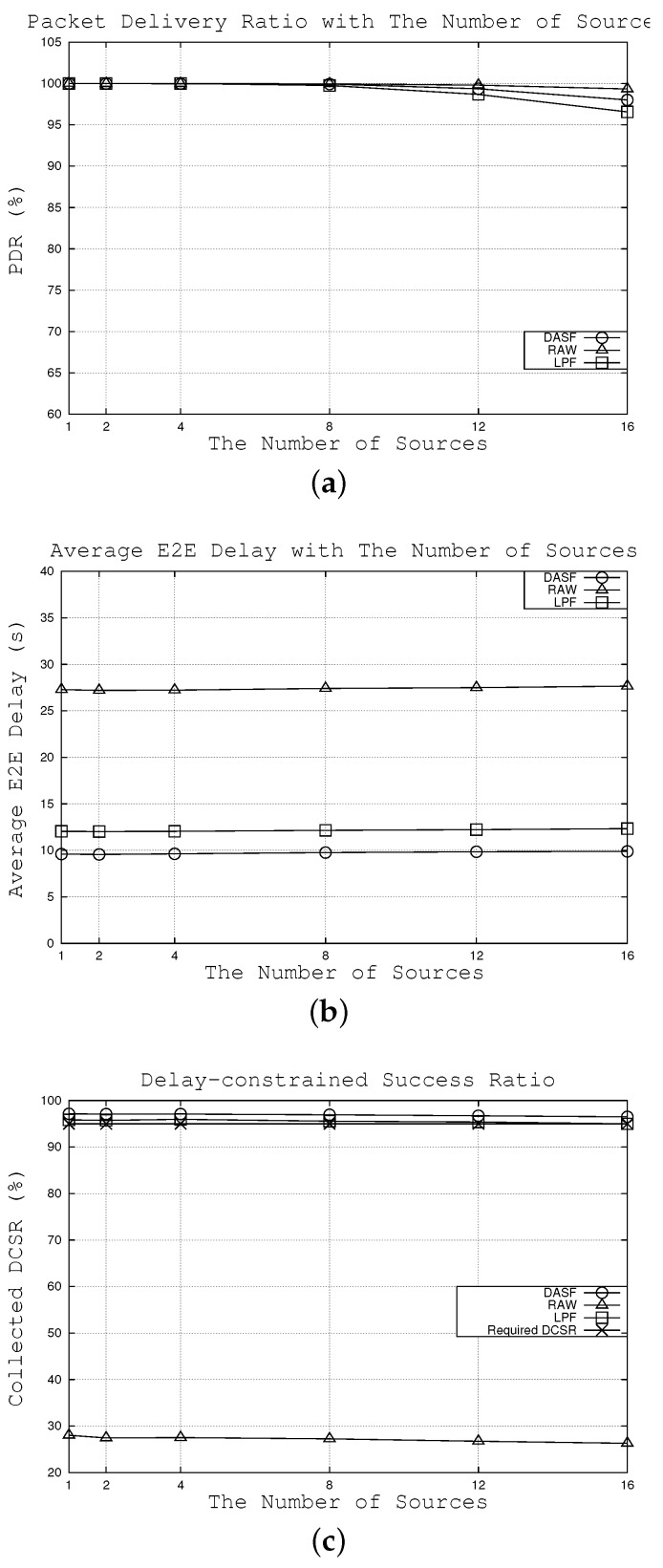
Effects of the number of sources. (**a**) effects on packet delivery ratio, (**b**) effects on average E2E delay, (**c**) effects on collected DCSR.

In this subsection, we can see how network performance is achieved when passing the number of nodes that generate the data packet whenever an event occurs from one to 16 sources. The duty cycle interval is calculated as 18.51 s using Equation (17) according to the variety of network parameters, such as the number of nodes, node density and delay requirement. Figure 8a shows that when the number of sources increases, PDR tends to decrease. However, similar to Subsection 6.2.2, LPF shows the strongest drop in PDR among the three considered algorithms. For example, when the number of source nodes is 16, PDR is 96.6%, 98.0% and 99.3% for LPF, DASF and RAW algorithms, respectively.

In terms of average E2E delay, Figure 8b shows that the average E2E delay in the three algorithms stays relatively flat, but DASF can achieve the lowest packet latency among the three considered algorithms. This is attributed to the fact that the duty cycle interval, not the number of sources, plays an important role in the average E2E delay. As a result, packet latency remains relatively consistent with the different number of sources. For instance, when there are eight source nodes, the average E2E delay is around 9.76 s under our algorithm compared to approximately 12.14 s and 27.40 s under LPF and RAW, respectively.

Moreover, as can be seen from Figure 8c, our work and LPF can satisfy the delay requirement, whereas RAW does not meet the required DCSR. Note that DASF achieves the highest collected DCSR in all cases. For example, when there are eight source nodes, 96.9% of the packets can be transmitted to the sink within 20 s compared to 95.5% and 27.2% with LPF and RAW, respectively.

According to these results, we can conclude that the proposed algorithm not only meets the required latency given the delay-constrained success ratio, but also achieves low delay compared to existing algorithms. In addition, the DASF algorithm can achieve a high packet delivery ratio over different network parameters.

## 7. Conclusion and Future Work

There are a number of applications of WSNs that require low energy consumption and a specific packet delay, e.g., forest fire detection or intruder targeting. This paper has addressed the problems of maximizing the duty cycle interval in a duty-cycled WSN, such that packets are forwarded to the sink within a delay bound with a given probability of success. A novel algorithm, namely DASF, was proposed, which enables the operator to adjust the duty cycle interval to meet the different delay requirements and evaluate the needed sources in a duty-cycled WSN. In addition, our algorithm makes a forwarding decision based on local information and does not require time synchronization between nodes.

In DASF, to maximize the value of the duty cycle interval, we evaluated the E2E delay’s distribution as a function of duty cycle interval and other network parameters, such as the number of nodes, the total area, the communication range of a node, and so forth. Therefore, we can adjust the duty cycle interval to satisfy a given delay requirement. Moreover, DASF also allows the operator to predict the needed sources, such as the total number of nodes for a certain area in a WSN. The simulation results show that our work can adjust the duty cycle interval with a variety of requirement changes to satisfy the required DCSR; and DASF can also reach a high PDR and lower average E2E delay compared to other algorithms.
